# Biomarkers associated with admission rupture status of intracranial aneurysms: a combined analysis of morphological, inflammatory and coagulation indicators

**DOI:** 10.3389/fneur.2026.1934306

**Published:** 2026-10-21

**Authors:** Pan Liu, Yunxiang Wang, Zhiqiang Zhao, Xiao Liu, Quan Ren, Zhengang Tang, Junjie Luo

**Affiliations:** Department of Neurosurgery, Renmin Hospital, Hubei University of Medicine, Shiyan, Hubei, China

**Keywords:** coagulation biomarkers, inflammatory biomarkers, intracranial aneurysm, nomogram, rupture status, subarachnoid hemorrhage

## Abstract

**Background:**

Spontaneous subarachnoid hemorrhage (SAH) is predominantly caused by intracranial aneurysm (IA) rupture. As a critical neurological emergency with rapid clinical deterioration, SAH is life-threatening and associated with high mortality and disability rates. Early and accurate discrimination of ruptured status is essential for the emergency management of intracranial aneurysms.

**Objective:**

This study aimed to screen admission inflammatory and coagulation biomarkers associated with IA rupture status and construct a comprehensive diagnostic model to distinguish ruptured from unruptured intracranial aneurysms.

**Methods:**

A single-center retrospective analysis was conducted on 167 patients with intracranial aneurysms admitted to the Department of Neurosurgery from January 2020 to September 2025. Patients were divided into a ruptured group (119 cases) and an unruptured group (48 cases) based on intraoperative and imaging findings. Baseline clinical, morphological, hematological and coagulation data were collected. LASSO regression was used for preliminary variable screening. Firth-penalized multivariate logistic regression was further applied to screen independent correlates of aneurysm rupture status and eliminate collinear and insignificant indicators. Finally, a three-variable combined model incorporating aneurysm morphology, white blood cell count, and D-dimer level was established. ROC curve analysis, 1,000-bootstrap calibration curve, and decision curve analysis (DCA) were performed to evaluate the discriminative ability, calibration consistency, and clinical net benefit of the model.

**Results:**

Irregular aneurysm morphology (OR = 37.13, 95%CI:7.26–189.75, *p* < 0.001), elevated white blood cell count (OR = 15.34, 95%CI:2.12–110.94, *p* < 0.001), and increased D-dimer level (OR = 12.27, 95%CI:2.26–66.70, *p* < 0.001) were independent biomarkers associated with ruptured IA status. After excluding collinear neutrophil counts, the final combined model achieved an AUC of 0.96. Bootstrap internal validation demonstrated excellent calibration with a mean absolute error of 0.017. DCA verified that the model yielded favorable clinical net benefit within a reasonable threshold probability range.

**Conclusion:**

Irregular aneurysm morphology, elevated white blood cell count, and increased D-dimer level at admission are independent biomarkers distinguishing ruptured from unruptured intracranial aneurysms. The multi-index combined model shows good discriminative performance, stable calibration, and promising clinical utility, which can provide objective auxiliary evidence for the rapid identification of aneurysm rupture status in neurosurgical emergency settings.

## 1 Introduction

Intracranial aneurysm (IA) refers to the pathological focal dilatation of intracranial arterial wall. The prevalence of unruptured IA in the general population is approximately 6.6%, and IA rupture constitutes the leading cause of spontaneous subarachnoid hemorrhage, a catastrophic condition associated with high mortality and disability rates (1, 2). At present, clinicians mainly rely on morphological imaging features (aneurysm size, location and shape) and the PHASES score to evaluate the risk of aneurysm rupture in patients with unruptured IA (3). However, these assessment systems only incorporate demographic and anatomical indicators while ignoring the biological pathological changes underlying aneurysm rupture, resulting in evident limitations in risk evaluation (4).

Existing pathological and clinical studies have verified that chronic inflammatory infiltration of vessel walls and dysregulated coagulation-fibrinolysis systems serve as core mechanisms contributing to arterial wall injury and subsequent aneurysm rupture (5, 6). Peripheral inflammatory markers such as white blood cell count can reflect systemic inflammatory burden and cause persistent endothelial damage; elevated D-dimer and other coagulation biomarkers indicate localized microvascular injury, and these indicators have been reported to be closely correlated with aneurysm rupture status (7, 8). It should be noted that blood biomarkers measured upon admission may also be affected by acute systemic stress response secondary to subarachnoid hemorrhage. Nevertheless, most previous studies merely analyzed inflammatory or coagulation markers separately, and few systematically explored correlates of IA rupture status by combining morphological, inflammatory and coagulation indicators from multiple dimensions (9).

Single morphological parameters fail to fully elaborate the complicated triggers of aneurysm-related clinical presentation, and multi-indicator joint analysis carries vital clinical significance for distinguishing ruptured from unruptured IAs in emergency settings. This study integrates aneurysm morphological features, routine peripheral inflammatory and coagulation biomarkers. Through intergroup comparison and regression analysis, we screen independent biomarkers associated with admission rupture status of IA and clarify the correlations between inflammatory/coagulation markers and aneurysm rupture status, so as to provide objective clinical evidence for emergency identification of ruptured intracranial aneurysms.

## 2 Materials and methods

### 2.1 Study design

This single-center retrospective study collected demographic data, medical history, imaging morphological characteristics of intracranial aneurysms (IA), as well as admission inflammatory and coagulation laboratory indicators of IA patients, and screened independent biomarkers associated with admission IA rupture status through multi-dimensional combined indicators. All procedures of this study were approved by the Institutional Review Board of our hospital (Approval no: SYSRMYY-KYXS-2026-025). Data collection and statistical analysis strictly complied with the medical research norms of the Declaration of Helsinki. The Institutional Review Board waived the requirement for written informed consent for this retrospective study.

### 2.2 Study subjects

#### 2.2.1 Inclusion criteria

1. Intracranial aneurysm confirmed by DSA, CTA or MRA, including ruptured and unruptured aneurysms;
2. Receiving microsurgical clipping or endovascular embolization;
3. Complete electronic medical records, imaging data and initial admission laboratory results of inflammatory and coagulation markers;
4. Age ≥ 18 years old, regardless of gender.

#### 2.2.2 Exclusion criteria

1. Combined with other cerebrovascular lesions such as infectious aneurysms, dissecting aneurysms, pseudoaneurysms, cerebral arteriovenous fistulas and moyamoya disease;
2. Patients receiving conservative treatment without surgical or endovascular intervention;
3. Critical missing data in demographic, imaging, inflammatory or coagulation laboratory indicators that cannot support statistical comparison;
4. Systemic diseases interfering with peripheral inflammatory and coagulation markers, including malignant tumors, primary hematological diseases and severe liver and renal dysfunction;
5. Previous history of surgical or endovascular treatment for intracranial aneurysms.

#### 2.2.3 Case screening process

Inpatients diagnosed with suspected or definite intracranial aneurysms from January 2020 to September 2025 in the Department of Neurosurgery were retrospectively enrolled. A total of 333 cases were initially screened. After excluding patients meeting the exclusion criteria, 167 subjects with complete data were finally included.

#### 2.2.4 Grouping criteria

All patients were divided into ruptured group (119 cases) and unruptured group (48 cases) according to aneurysm rupture status identified by preoperative CTA/MRA/DSA and intraoperative findings. Rupture judgment standard: Spontaneous subarachnoid hemorrhage corresponding to the aneurysm, ruptured defects on the aneurysm wall shown by imaging, or intraoperative visible aneurysm rupture and hemorrhage. Baseline comparison between the two groups was performed to screen biomarkers correlated with IA rupture status upon admission.

### 2.3 Data collection

All data were extracted from the electronic medical record system of our hospital. The first venous blood samples collected on admission were uniformly adopted for laboratory detection. Collected data covered demographics, underlying medical history, imaging morphological features of aneurysms, peripheral inflammatory and coagulation indicators.

1. Demographic data: gender and age; patients were divided into < 60 years group and ≥ 60 years group with 60 years old as the cut-off value.
2. Medical history: smoking history, drinking history, hypertension, diabetes and coronary heart disease.
3. Imaging characteristics of aneurysms (judged by associate chief physicians and above via imaging reports or surgical records): aneurysm location (anterior circulation / posterior circulation), morphology (regular / irregular), single / multiple aneurysms, maximum diameter (> 10 mm / ≤ 10 mm), aspect ratio (> 1.5 / ≤ 1.5), intracranial arteriosclerosis, arterial stenosis (stenosis ≥ 50% defined as arterial stenosis).
4. Peripheral inflammatory indicators (abnormal values defined by reference range of hospital clinical laboratory): elevated white blood cell count (> 9.5 × 10^9^/L), elevated neutrophil count (> 6.3 × 10^9^/L), decreased lymphocyte count (< 1.0 × 10^9^/L).
5. Coagulation function indicators (abnormal values defined by reference range of hospital clinical laboratory): elevated D-dimer (> 0.5 mg/L), prolonged APTT (> 37 s), prolonged PT (> 13.5 s), prolonged TT (> 19 s).

### 2.4 Statistical analysis

Categorical data were described as case number (percentage), and intergroup comparisons were performed using the *χ*^2^ test. Measurement data were expressed as mean ± standard deviation. All available clinical, imaging, and laboratory variables were initially included for screening. LASSO regression with 10-fold cross-validation was performed to remove redundant variables and mitigate multicollinearity. Considering the limited sample size and sparse data distribution, Firth-penalized multivariate logistic regression was further applied to identify independent biomarkers associated with IA rupture status. Variance inflation factor (VIF) was calculated to evaluate collinearity. Neutrophil count with high collinearity and insignificant variables were excluded, and the final model incorporated only irregular aneurysm morphology, elevated white blood cell count, and increased D-dimer level. A nomogram was constructed to visualize the contribution of each indicator. Bootstrap internal validation with 1,000 resamples was conducted to assess model calibration, and the mean absolute error (MAE) was calculated. ROC curve analysis was used to evaluate discriminative ability, and decision curve analysis (DCA) was performed to assess clinical net benefit. Notably, all blood samples were collected at admission after aneurysm rupture occurred in ruptured cases. The detected inflammatory and coagulation biomarkers may reflect acute systemic stress response secondary to subarachnoid hemorrhage rather than pre-rupture baseline risk factors. Therefore, this study aimed to identify admission biomarkers for distinguishing ruptured and unruptured IAs, rather than predicting long-term prospective rupture risk. Statistical analyses were performed using R 4.4.1. A two-tailed *p* < 0.05 was considered statistically significant.

## 3 Results

### 3.1 Baseline characteristics of study population

All participants were divided into ruptured and unruptured groups based on preoperative imaging findings. Aneurysm morphological features and admission laboratory biomarkers were compared between the two groups, while post-rupture clinical manifestations were excluded to avoid temporal confounding bias. No significant intergroup differences were observed in gender, age, hypertension, coronary heart disease, smoking, drinking history or aneurysm location (all *p* > 0.05). A statistically significant difference was detected in the prevalence of diabetes mellitus: the rate reached 16.67% in the unruptured group versus only 5.04% in the ruptured group (*p* = 0.014). In terms of aneurysm morphology, aneurysm irregularity differed markedly between groups with a standardized mean difference of 1.98 (*p* < 0.001). Irregular aneurysms accounted for 92.44% of all lesions in the ruptured cohort. The proportion of multiple aneurysms was also significantly higher in ruptured patients (40.34%) compared with unruptured counterparts (20.83%, *p* = 0.017). Laboratory examinations revealed that the ruptured group had significantly higher proportions of patients with elevated white blood cell count, reduced lymphocyte count and increased D-dimer (all *p* < 0.001). No intergroup differences were found in aneurysm diameter, aspect ratio, cerebrovascular lesions or other coagulation parameters (all *p* > 0.05; See Table 1).

**Table 1:** Comparison of general clinical baseline data between ruptured and unruptured intracranial aneurysm groups.

	Unruptured	Ruptured	Standardize diff.	*p*-value	*p*-value*
Gender			0.10 (−0.24, 0.43)	0.560	–
Female	30 (62.50%)	80 (67.23%)			
Male	18 (37.50%)	39 (32.77%)			
Years			0.13 (−0.21, 0.46)	0.459	–
<60	24 (50.00%)	67 (56.30%)			
≥60	24 (50.00%)	52 (43.70%)			
Hypertension			0.04 (−0.29, 0.38)	0.796	–
No	20 (41.67%)	47 (39.50%)			
Yes	28 (58.33%)	72 (60.50%)			
Diabetes			0.38 (0.04, 0.72)	0.014	–
No	40 (83.33%)	113 (94.96%)			
Yes	8 (16.67%)	6 (5.04%)			
CHD			0.22 (−0.11, 0.56)	0.176	–
No	39 (81.25%)	106 (89.08%)			
Yes	9 (18.75%)	13 (10.92%)			
Smoking			0.00 (−0.33, 0.34)	0.983	–
No	40 (83.33%)	99 (83.19%)			
Yes	8 (16.67%)	20 (16.81%)			
Drinking			0.17 (−0.17, 0.50)	0.349	–
No	42 (87.50%)	97 (81.51%)			
Yes	6 (12.50%)	22 (18.49%)			

Table 1 Baseline characteristics of patients with ruptured versus unruptured intracranial aneurysms.

### 3.2 Variable selection via LASSO regression model

All candidate indicators correlated with intracranial aneurysm rupture were incorporated into the LASSO regression model. Variables related to postoperative outcomes were excluded in advance to prevent information leakage and reverse causality. With aneurysm rupture defined as the outcome variable, 10-fold cross-validation was performed for LASSO regression. The optimal penalty coefficient λ was determined as the lambda value corresponding to the minimum penalty coefficient plus one standard deviation. Seven independent variables with non-zero coefficients were ultimately screened out: diabetes mellitus, irregular aneurysm morphology, headache, elevated white blood cell count, elevated neutrophil count, decreased lymphocyte count, and elevated D-dimer level (see Figure 1 for details; Tables 2, 3).

**Figure 1:**
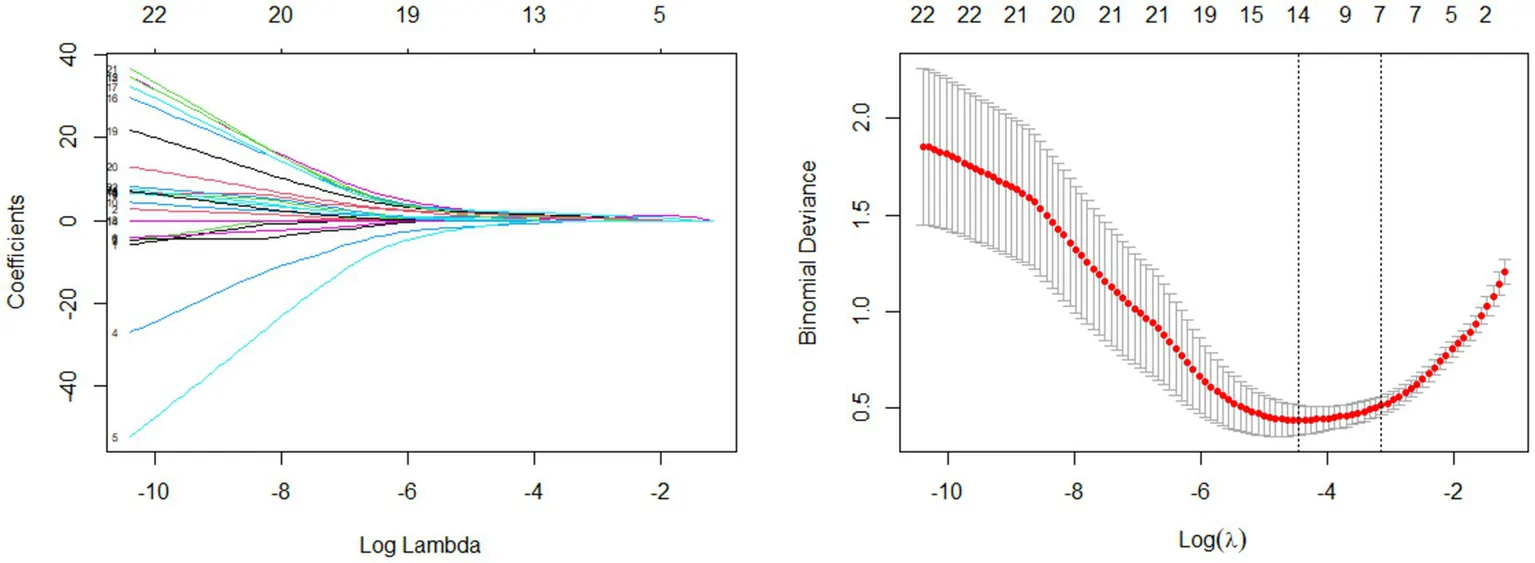
**(A)** LASSO coefficient profile of the seven independent variables. **(B)** 10-fold cross-validation results of the LASSO model. Seven variables: diabetes mellitus, irregular aneurysm morphology, headache, elevated white blood cell count, elevated neutrophil count, decreased lymphocyte count, elevated D-dimer level. In this figure Variable screening results for intracranial aneurysm rupture based on LASSO regression.

**Table 2:** Comparison of morphological characteristics of intracranial aneurysms between ruptured and unruptured groups.

	Unruptured	Ruptured	Standardize diff.	*p*-value	*p*-value*
Aneurysm location			0.02 (−0.32, 0.35)	0.924	–
Anterior circulation	43 (89.58%)	106 (89.08%)			
Posterior circulation	5 (10.42%)	13 (10.92%)			
Aneurysm>10 mm			0.25 (−0.08, 0.59)	0.163	–
No	43 (89.58%)	96 (80.67%)			
Yes	5 (10.42%)	23 (19.33%)			
Aneurysm irregularity			1.98 (1.58, 2.38)	<0.001	–
No	37 (77.08%)	9 (7.56%)			
Yes	11 (22.92%)	110 (92.44%)			
Multiple aneurysms			0.43 (0.09, 0.77)	0.017	–
No	38 (79.17%)	71 (59.66%)			
Yes	10 (20.83%)	48 (40.34%)			
AR of the aneurysm>1.5			0.25 (−0.08, 0.59)	0.163	–
No	43 (89.58%)	96 (80.67%)			
Yes	5 (10.42%)	23 (19.33%)			
Aneurysm stenosis			0.18 (−0.15, 0.52)	0.277	–
No	28 (58.33%)	80 (67.23%)			
Yes	20 (41.67%)	39 (32.77%)			
Arteriosclerosis			0.23 (−0.10, 0.57)	0.170	–
No	22 (45.83%)	41 (34.45%)			
Yes	26 (54.17%)	78 (65.55%)			

**Table 3:** Comparison of clinical manifestations and related indicators between ruptured and unruptured intracranial aneurysm groups.

	Unruptured	Ruptured	Standardize diff.	*p*-value	*p*-value*
Headache			1.11 (0.76, 1.47)	<0.001	–
No	31 (64.58%)	20 (16.81%)			
Yes	17 (35.42%)	99 (83.19%)			
WBC			2.00 (1.61, 2.40)	<0.001	–
No	46 (95.83%)	32 (26.89%)			
Yes	2 (4.17%)	87 (73.11%)			
Neutrophil			2.02 (1.62, 2.42)	<0.001	–
No	41 (85.42%)	17 (14.29%)			
Yes	7 (14.58%)	102 (85.71%)			
Lymphocyte			1.76 (1.38, 2.15)	<0.001	–
No	39 (81.25%)	18 (15.13%)			
Yes	9 (18.75%)	101 (84.87%)			
D-dimer			1.26 (0.90, 1.62)	<0.001	–
No	45 (93.75%)	53 (44.54%)			
Yes	3 (6.25%)	66 (55.46%)			
APTT			0.11 (−0.22, 0.45)	0.529	–
No	46 (95.83%)	111 (93.28%)			
Yes	2 (4.17%)	8 (6.72%)			
PT			0.15 (−0.18, 0.49)	0.387	–
No	40 (83.33%)	92 (77.31%)			
Yes	8 (16.67%)	27 (22.69%)			
TT			0.09 (−0.24, 0.43)	0.593	–
No	43 (89.58%)	103 (86.55%)			
Yes	5 (10.42%)	16 (13.45%)			

APTT, activated partial thromboplastin time; PT, prothrombin time; TT, thrombin time. Standardized mean difference (SMD) for categorical variables is provided to quantify the magnitude of inter-group imbalance. Statistical inference for group differences is based on *χ*^2^ test or Fisher’s exact test *p*-values.

### 3.3 Establishment of multivariate logistic regression model

Variables screened by LASSO regression for distinguishing aneurysm rupture status were incorporated into the multivariate logistic regression model. Four variables were finally identified to be significantly associated with aneurysm rupture: irregular aneurysm morphology (OR = 37.13, 95%CI: 7.26–189.75, *p* < 0.001), elevated white blood cell count (OR = 15.34, 95%CI: 2.12–110.94, *p* < 0.001), elevated neutrophil count (OR = 9.69, 95%CI: 1.98–47.41, *p* < 0.001), and elevated D-dimer level (OR = 12.27, 95%CI: 2.26–66.70, *p* < 0.001). Detailed results are shown in Table 4.

**Table 4:** Multivariate logistic regression analysis of indicators associated with intracranial aneurysm rupture status.

Variable	*β*	SE	Wald	OR	95% CI	*p*-value
Irregular aneurysm	3.61	0.83	18.86	37.13	7.26–189.75	<0.001
Elevated WBC	2.73	1.01	7.32	15.34	2.12–110.94	<0.001
Elevated neutrophil	2.27	0.81	7.86	9.69	1.98–47.41	<0.001
D-dimer	2.51	0.86	8.43	12.27	2.26–66.70	<0.001
Diabetes	−2.11	1.90	1.23	0.12	0.00–5.07	0.268
Headache	1.14	0.77	2.22	3.13	0.69–14.10	0.137
lymphocyte	2.06	1.76	1.37	7.81	0.25–247.23	0.243

The wide 95% confidence intervals for inflammatory markers may reflect quasi-complete separation, as elevated WBC and neutrophil counts were rare in the unruptured group.

### 3.4 Multicollinearity assessment

Variance inflation factor (VIF) was calculated to evaluate multicollinearity among the predictors. Elevated neutrophil count (VIF = 6.99) and lymphocyte count (VIF = 5.71) exhibited VIF > 5, indicating moderate collinearity. Since lymphocyte was not statistically significant in the multivariable model (*p* = 0.243), it was excluded from further analysis. The collinearity between WBC and neutrophil count was attributable to neutrophils constituting the major subset of leukocytes. Detailed results are shown in Table 5.

**Table 5:** Multicollinearity assessment using VIF.

Variable	Tolerance	VIF
Diabetes	0.968	1.034
Irregular aneurysm	0.651	1.535
Headache	0.762	1.312
Elevated WBC	0.499	2.005
Elevated neutrophil	0.143	6.987
lymphocyte	0.175	5.712
D-dimer	0.828	1.208

### 3.5 Firth’s penalized regression and variable selection

Given the wide confidence intervals observed in the conventional model, suggesting potential quasi-complete separation and small-sample bias, Firth’s penalized logistic regression was performed as a sensitivity analysis. In the Firth model, elevated neutrophil count lost statistical significance (OR = 6.15, 95%CI: 0.86–44.09, *p* = 0.071), whereas the other three variables remained significant: irregular aneurysm morphology (OR = 24.70, 95%CI: 3.67–166.02, *p* = 0.001), elevated WBC (OR = 17.19, 95%CI: 1.44–205.17, *p* = 0.025), and elevated D-dimer (OR = 15.53, 95%CI: 1.94–124.49, *p* = 0.010). A subsequent model excluding WBC also yielded non-significant results for neutrophil count (*p* > 0.05). Therefore, neutrophil count was excluded from the final model, and three independent predictors were retained: irregular aneurysm morphology, elevated WBC, and elevated D-dimer. The conventional and Firth regression results are compared in Table 6.

**Table 6:** Comparison of conventional and firth’s penalized logistic regression.

Variable	Conventional			Firth		
	OR	95% CI	*p*	OR	95% CI	*p*
Irregular aneurysm	37.13	7.26–189.75	<0.001	24.70	3.67–166.02	0.001
Elevated WBC	15.34	2.12–110.94	<0.001	17.19	1.44–205.17	0.025
Elevated neutrophil	9.69	1.98–47.41	<0.001	6.15	0.86–44.09	0.071
Elevated D-dimer	12.27	2.26–66.70	<0.001	15.53	1.94–124.49	0.010

Neutrophil count lost statistical significance in the Firth model and was excluded from the final model.

### 3.6 Nomogram of factors associated with intracranial aneurysm rupture

The independent risk factors for aneurysm rupture, including irregular aneurysm morphology, elevated white blood cell count, and increased D-dimer level, were incorporated into the final model. A nomogram (Figure 2) was constructed to visually quantify the individual contribution of each significant predictor to the risk of intracranial aneurysm rupture.

**Figure 2:**
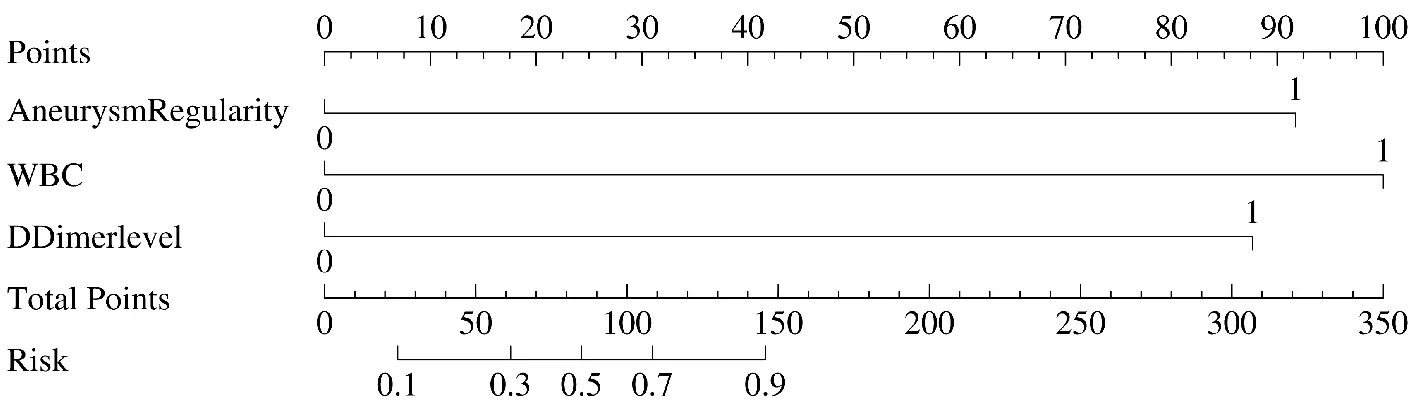
Estimate the probability of intracranial aneurysm rupture. The nomogram was established based on multivariate logistic regression, incorporating irregular aneurysm morphology, elevated white blood cell count and increased D-dimer level. Scores were assigned to each variable via the nomogram to estimate the probability of intracranial aneurysm rupture.

### 3.7 Validation of nomogram performance for intracranial aneurysm rupture

We constructed three nested models with incremental predictors: ModelA1 included only irregular aneurysm morphology; ModelA2 incorporated irregular aneurysm morphology and increased D-dimer level; ModelA3 (final nomogram model) further added elevated white blood cell count. The AUC values for ModelA1, ModelA2 and ModelA3 were 0.85 (95%CI:0.78–0.91), 0.91 (95%CI:0.87–0.96) and 0.96 (95%CI:0.94–0.99), respectively. The final three-predictor nomogram exhibited numerically higher discriminative ability compared with the other two nested models. The nomogram was established based on Firth-penalized multivariate logistic regression. Scores were assigned to each variable via the nomogram to estimate the probability of intracranial aneurysm rupture. Bootstrap internal validation with 1,000 resamples was performed to assess model calibration; the mean absolute error (MAE) was 0.017, demonstrating favorable consistency between model-predicted rupture risk and actual observed outcomes. Decision-curve analysis further suggested that the nomogram provided favorable net clinical benefit within a reasonable range of threshold probabilities (Figure 3).

**Figure 3:**
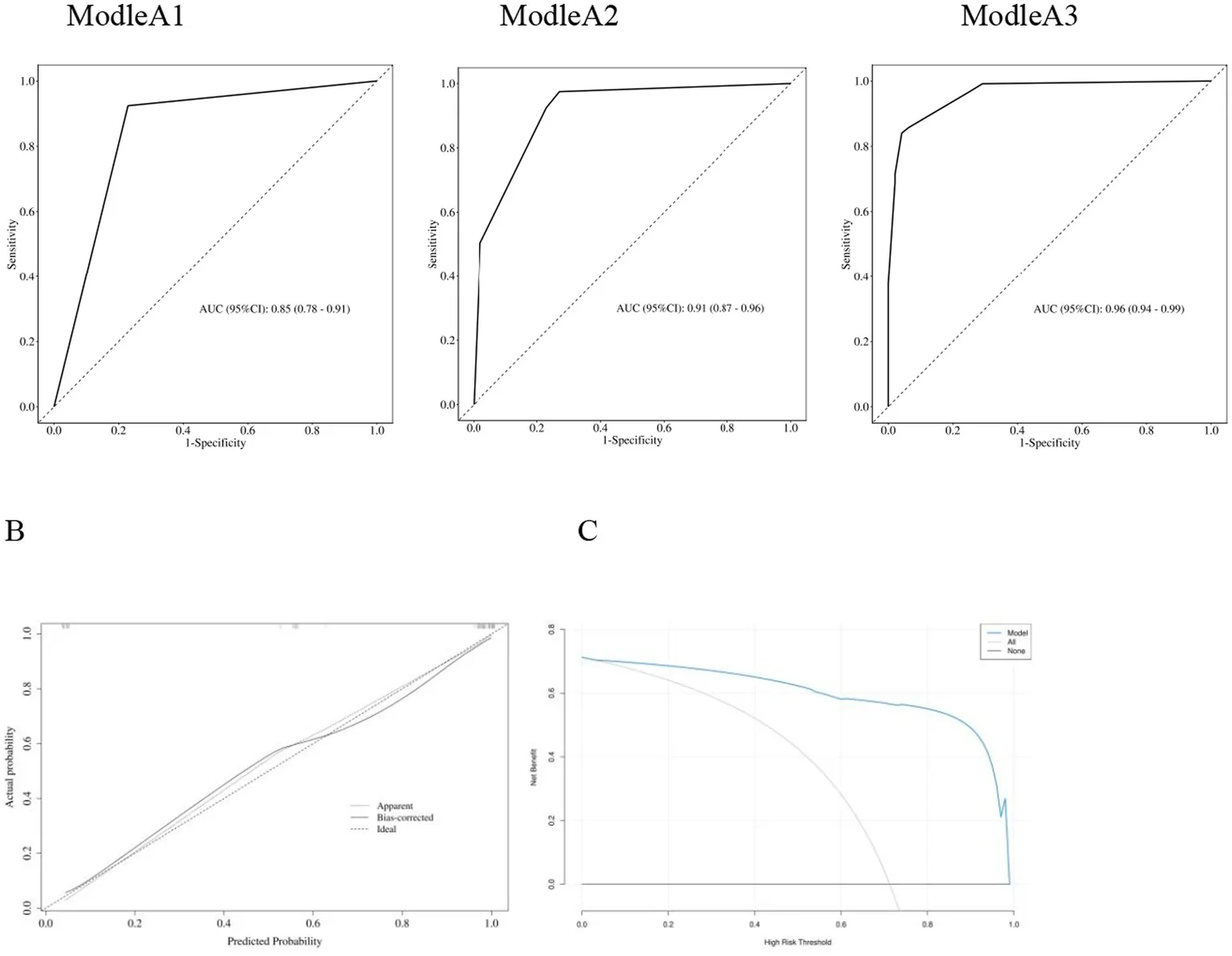
Validation results of the nomogram for identifying intracranial aneurysm admission rupture status. **(A)** ROC curves of three nested prediction models. The AUC of the final three-predictor model was 0.96, showing excellent discriminative performance. **(B)** The calibration curve (CC) revealed high consistency between predicted and actual rupture probabilities with a mean absolute error of 0.017. A mean absolute error (MAE) < 0.05 is generally regarded as acceptable for model calibration; the MAE of 0.017 in this study indicates good calibration performance. **(C)** Decision-curve analysis (DCA) demonstrated favorable clinical utility of the model across reasonable threshold probabilities.

## 4 Discussion

Intracranial aneurysm (IA) is a common high-risk cerebrovascular disorder. Rupture of IA leads to spontaneous subarachnoid hemorrhage and intraventricular hematoma, which rapidly triggers life-threatening complications such as acute intracranial hypertension and cerebral hernia, accompanied by high mortality and disability rates. As aneurysm rupture presents as an acute emergency, rapid and accurate identification of rupture status-related biomarkers is critical for emergency assessment and clinical decision-making. Existing studies have demonstrated that the occurrence and progression of IA rupture are not driven by a single factor, but represent a pathological process synergistically affected by anatomical morphology, systemic inflammation, and coagulation function. Nevertheless, a comprehensive and systematic multi-index evaluation system combining morphological features and serological biomarkers for distinguishing IA rupture status has not yet been established (8, 10). Accordingly, this single-center retrospective study integrated demographic baseline data, aneurysm morphological features, and routine admission inflammatory and coagulation biomarkers to screen independent biomarkers associated with IA rupture status. After strict variable screening and collinearity elimination, irregular aneurysm morphology, elevated white blood cell count, and increased D-dimer level were finally identified as core correlated indicators, further elucidating the clinical value of inflammation-coagulation dysregulation in auxiliary identification of aneurysm rupture status.

### 4.1 Baseline demographic and clinical characteristics associated with IA rupture

A total of 167 patients were divided into ruptured and unruptured groups according to preoperative imaging-confirmed aneurysm rupture status to analyze the correlation between baseline clinical profiles and IA rupture status. Females accounted for the majority of the cohort (n = 110), consistent with previous epidemiological evidence indicating female gender as a demographic characteristic closely related to IA occurrence (11, 12). Statistical analysis revealed no significant association between age and IA rupture status (*p* = 0.459), in line with multiple prior studies suggesting age cannot serve as a valid indicator for distinguishing aneurysm rupture status (13, 14). Anatomically, most aneurysms were located in the anterior circulation (n = 149) and were mostly small-to-medium sized. This distribution pattern is consistent with large-sample epidemiological surveys and matches the clinical epidemiological characteristics of IA (15).

Notably, postoperative and post-rupture clinical manifestations were excluded from baseline comparison in this study to avoid temporal confounding bias and information leakage. Clinical symptoms such as headache and disturbed consciousness only occur secondary to subarachnoid hemorrhage after aneurysm rupture, which reflect the severity of acute hemorrhagic injury rather than pre-existing baseline characteristics associated with rupture susceptibility. Therefore, these indicators cannot be used as biomarkers for judging preoperative rupture risk. Baseline laboratory analyses focused on admission inflammatory and coagulation biomarkers with stable baseline attributes. The results showed that patients with ruptured IA exhibited significantly elevated admission white blood cell count and D-dimer level, while routine coagulation indices (PT, APTT) showed no significant intergroup differences. This finding indicates that inflammatory activation and coagulation abnormal changes are core serological manifestations correlated with IA rupture status, and the asynchronous alteration of inflammation and conventional coagulation indicators further suggests complex interactive regulatory mechanisms between inflammatory and coagulation-fibrinolysis systems after IA injury, providing clinical evidence for exploring inflammation-coagulation cascades underlying aneurysm rupture status (12).

### 4.2 Value of combined inflammatory, coagulation and morphological markers as risk factors for IA rupture

After preliminary variable screening using LASSO regression and further collinearity elimination and optimization via Firth-penalized logistic regression, irregular aneurysm morphology, elevated white blood cell count, and increased D-dimer level were finally identified as independent biomarkers associated with IA rupture status. Different from previous single-index evaluations, this multi-dimensional combination of morphological and admission serological indicators reflects the comprehensive clinical characteristics of ruptured IAs, demonstrating that aneurysm rupture status is closely correlated with anatomical structural abnormalities and systemic inflammation-coagulation alterations, The present findings provide novel auxiliary evidence for emergency identification of ruptured intracranial aneurysms.

Among the included indicators, irregular aneurysm morphology exhibited the most prominent correlation with rupture status. Irregular aneurysms usually present uneven wall thickness and focal structural weakness. Hemodynamic fluctuation leads to heterogeneous wall stress distribution, which renders structurally defective segments more susceptible to hemorrhage. In this study, the ruptured group had a significantly higher proportion of irregular aneurysms, which is consistent with previous imaging studies and further confirms that morphological abnormality is a core baseline feature closely related to aneurysm rupture (15).

Peripheral inflammatory biomarkers are readily available, non-invasive clinical indicators that reflect systemic inflammatory activation upon admission. Elevated white blood cell count was screened as an independent correlate of ruptured IA status in the present study. Increased systemic inflammatory response after subarachnoid hemorrhage can significantly elevate peripheral leukocyte levels, which partly explains the significant difference in inflammatory markers between the two groups. Combined with aneurysm morphological features, inflammatory biomarkers can effectively assist in distinguishing ruptured from unruptured IAs in emergency clinical scenarios (16).

This study also confirmed that elevated D-dimer level is an independent biomarker for IA rupture status, indicating that coagulation-fibrinolysis activation is closely related to aneurysm hemorrhagic status. As a sensitive marker of fibrinolytic system activation, increased D-dimer reflects localized microvascular injury and hyperfibrinolysis after aneurysm rupture. Compared with conventional PT and APTT indicators, D-dimer is more sensitive to acute coagulation-fibrinolysis disturbance after SAH and can better distinguish patients with ruptured IAs. Combined morphological, inflammatory, and coagulation markers can significantly improve the discriminatory efficiency for IA rupture status, providing reliable clinical evidence for auxiliary emergency judgment of aneurysm rupture (2, 17).

### 4.3 Strengths and limitations

This study integrated admission inflammatory-coagulation biomarkers and aneurysm morphological imaging features to analyze clinical indicators correlated with intracranial aneurysm rupture status, with several notable strengths. First, this multi-dimensional indicator system overcomes the inherent limitations of traditional morphology-only evaluation strategies. All enrolled serological and imaging indicators are routine, readily accessible, and cost-effective in clinical practice, rendering the combined model highly applicable for rapid auxiliary identification of aneurysm rupture status upon emergency admission. Second, the present study adopted rigorous and standardized statistical procedures. LASSO regression was applied to eliminate redundant variables and mitigate multicollinearity and overfitting bias. Firth-penalized multivariate logistic regression was further utilized to screen stable independent correlated indicators. Moreover, comprehensive model validation including ROC curve discrimination analysis, calibration curve assessment, and decision curve analysis was performed to systematically evaluate the model’s diagnostic performance and clinical net benefit, ensuring the credibility and stability of the analytical results.

Several unavoidable limitations of this study should be adequately acknowledged. First, this is a single-center retrospective study with all enrolled patients receiving surgical or endovascular intervention, which may introduce inherent selection bias and limit the external generalizability of the constructed multi-indicator model. Second, this study failed to incorporate hemodynamic parameters and vascular microstructural indices, which are proven pivotal in aneurysm pathological progression and rupture. In addition, all continuous laboratory indicators were dichotomized based on clinical reference ranges, which inevitably discarded partial quantitative information and nonlinear correlation characteristics, restricting the in-depth interpretation of the biological mechanism underlying aneurysm rupture status alteration. Third, all blood specimens were collected at admission after aneurysm onset in hemorrhagic patients. Single-point blood sampling cannot effectively distinguish whether the abnormal inflammatory and coagulation biomarkers represent potential pre-rupture baseline characteristics or acute systemic stress responses triggered by subarachnoid hemorrhage. Furthermore, external prospective cohort validation was absent in this study. Moreover, laboratory tests were not uniformly collected for all patients in this retrospective cohort, restricting the types of blood parameters available for analysis. In addition, we did not perform stratified subgroup analyses to explore the interactive effects of aneurysm morphology and hypertension on aneurysm rupture. Limited sample size of unruptured cases may render such subgroup analysis statistically unstable, and this issue could be better addressed in future studies with larger sample sizes.

In the future, multi-center, large-sample, and prospective longitudinal studies are warranted to further verify the stability, reproducibility, and clinical applicability of this multi-dimensional auxiliary diagnostic model for IA rupture status.

## 5 Conclusion

This study confirms that irregular aneurysm morphology, elevated white blood cell count, and increased D-dimer level are core independent biomarkers associated with intracranial aneurysm rupture status. Aneurysm morphological features reflect inherent structural wall defects, while admission inflammatory and coagulation indicators mirror acute systemic pathological alterations closely correlated with hemorrhagic status. This multi-dimensional framework integrating imaging morphological characteristics and routine biochemical markers enables comprehensive auxiliary identification of ruptured intracranial aneurysms. All included indicators are readily available on emergency admission, which can provide objective clinical evidence for rapid differentiation of aneurysm rupture status and assist clinicians in formulating individualized emergency management strategies for intracranial aneurysm patients.

## Data Availability

The raw data supporting the conclusions of this article will be made available by the authors, without undue reservation.
